# Secondhand Smoke Exposure and Health Services Use among Adolescent Current Smokers

**DOI:** 10.1371/journal.pone.0064322

**Published:** 2013-05-08

**Authors:** Man Ping Wang, Sai Yin Ho, Wing Sze Lo, Tai Hing Lam

**Affiliations:** School of Public Health, The University of Hong Kong, Pokfulam, Hong Kong; University of Liverpool, United Kingdom

## Abstract

**Background:**

To investigate the associations of secondhand smoke (SHS) exposure with medical consultation and hospitalisation among adolescents in Hong Kong.

**Methods:**

A total of 35827 secondary 1 (US grade 7) to secondary 5 students from 85 randomly selected schools completed an anonymous questionnaire on smoking, SHS at home, SHS outside home, medical consultation in the past 14 days, hospitalisation in the past 12 months, and socio-demographic characteristics. Current smoking was defined as any smoking in the past 30 days. SHS exposure was classified as none (reference), 1–4 and 5–7 days/week. Logistic regression yielded adjusted odds ratios (AORs) for medical consultation and hospitalisation in relation to SHS exposure at home and outside home in current smokers. Analyses were also done among never-smokers for comparison.

**Results:**

Among all students, 15.9% had medical consultation and 5.2% had been hospitalised. Any SHS exposure at home was associated with AORs (95% CI) for medical consultation and hospitalisation of 1.69 (1.14–2.51) and 2.85 (1.47–5.52) in current smokers, and 1.03 (0.91–1.15) and 1.25 (1.02–1.54) in never-smokers, respectively, (P<0.01 for interaction between smoking status and SHS exposure at home). SHS exposure outside home was generally not associated with medical consultation and hospitalisation in smokers and never-smokers.

**Conclusions:**

SHS exposure at home was associated with health services use among adolescent current smokers. Adolescent smokers should be aware of the harm of SHS in addition to that from their own smoking. The evidence helps health care professionals to advise adolescent smokers to avoid SHS exposure and stop smoking.

## Introduction

The harmful effects of secondhand smoke (SHS) exposure have been well established in never-smokers [1]. Substantial evidence links SHS exposure to medical services use in never-smoking children [2]–[5]. but only one study has reported such association among adult smokers [6]. Our literature search identified no studies on the relation between SHS exposure and health services use in smoking adolescents. The harmful effects of SHS exposure were generally neglected in smokers despite higher exposure [7]. Such heavy exposure is unsurprising as smokers tend to mix with smokers and expose themselves to SHS [8], [9]. We have reported that SHS exposure was linked to respiratory symptoms among adolescent and adult smokers [6], [10]. Although respiratory symptoms are common causes for medical services use among adolescents [11], it is unclear whether these adverse effects translate into medical services use by adolescent smokers.

In Hong Kong, medical services are easily accessible. Medical consultation is mainly provided by private general practitioners (66.3%) and government outpatient clinics (21.0%) [12]. Hospitalisation is mostly (90%) provided by public hospitals at low costs (US $13/day) [13]. Before 2007, smoking has been banned in shopping malls, cinemas, amusement game centers, schools and public transports, partially banned in restaurants and unrestricted in outdoor public places [14]. More comprehensive smokefree legislation was implemented in 2007 to cover most indoor public places and some outdoor places. These and subsequent increases in tobacco tax have seen adolescent smoking rates dropping from 9.6% in 2003/4 to about 3.4% in 2010 [15], which was lower than that in Western countries [16]. However, SHS exposures at home and outside home are still prevalent in Hong Kong [14], where homes are typically small and pavements narrow. We therefore examined the associations of SHS exposure at home and outside home with medical services use in adolescent smokers and non-smokers in 2003-4, before the 2007 smokefree legislation.

## Materials and Methods

### Ethics Statement

Ethical approval was granted by Institutional Review Board of the University of Hong Kong/Hospital Authority Hong Kong West Cluster (IRB). Informed consent was obtained from the schools, which acted in loco parentis for the students. The IRB approved the use of informed consent from schools instead of parental informed consent for minor participants in this survey. Invitation letter describing the details of the survey and voluntary participation were sent to the parents. Local practice for surveys does not require written consent, and participation constitutes consent. The voluntary basis of the study was clearly explained on the questionnaires and students can decide whether to participate in the study.

### Sampling

A Youth Smoking Survey was conducted among secondary 1 (US grade 7) to secondary 5 students (age 11–19) using 2-stage random sampling as in the Global Youth Tobacco Survey (GYTS) [16]. All secondary 1 students and 2 classes of each upper grade from 85 randomly selected schools (63.9% responded) were surveyed with a high student response rate (98%). The core items of the Chinese version of GYTS questionnaire was administered in classrooms. Teachers maintained classroom order and let students answer independently. To ensure candid reporting, completed anonymous answer sheets were immediately collected and sealed by our research staff. Detailed survey methods have been reported elsewhere [10], [17], [18].

### Measurement

Students reported their smoking status, years of smoking, average number of cigarettes smoked per smoking day in the past 30 days, SHS exposures in the past 7 days, number of co-residing smokers, alcohol drinking (daily, weekly, monthly or never), drug abuse (ever or never), demographic characteristics, and socioeconomic status (SES) indicators of highest parental education and housing type. Current smoking (N = 2420) was defined as any smoking in the past 30 days. Students reported the number of days in the past 7 days that someone smoked near at home and outside home in two separate questions of “In the past 7 days, how many days have someone smoked near you at home?” and “In the past 7 days, how many days have someone smoked near you outside home?”. SHS exposure was categorized as none (reference), 1–4 days/week and 5–7 days/week so as to compare with the findings of our previous study.[10] Medical consultation of Western and Chinese medical practitioners in the past 14 days and hospitalisation in the past 12 months were reported by the students. Medical consultation was defined as having any Western or Chinese medical consultation in the past 14 days, and hospitalisation was defined as any hospital admission in the past 12 months.

### Statistical analysis

After excluding 718 (2%) questionnaires with response sets (obvious answering patterns in answer sheets) or excessive missing data (more than 50% missing value), 35827 students (98%) remained for analysis using STATA 9.2. Logistic regression yielded adjusted odds ratios (AORs) for medical consultation and hospitalisation in relation to SHS exposure at home and outside home adjusting for each other and sex, age (in years), highest parental education, housing type, and school clustering effects. The AORs were calculated separately for current smokers and never-smokers. The linear associations between SHS exposure and medical services use were tested by treating SHS exposure at home and outside home as continuous variables (p for trend). Potential effect modification was tested using an interaction term of SHS*smoking status adjusting for socio-demographic characteristic and school clustering effects. For current smokers, years of smoking and amount smoked per day were also adjusted for. To reduce any confounding effects of SHS exposure outside home, students who reported >2 days/week of such exposure (46.9%) were excluded from the calculation of AORs for SHS exposure at home. Residual confounding effects of SHS outside home (1 or 2 days/week) were also adjusted for. Similarly, students who were exposed to SHS at home for >2 days/week (45.5%) were excluded from the calculation of AORs for SHS exposure outside home.

## Results

### Descriptive data

Table 1 shows that medical consultation was reported by 15.9% of students with a higher prevalence in girls; hospitalisation (5.2%) was less common, especially in girls. The highest prevalence of medical consultation and hospitalisation was observed among students with the lowest socioeconomic status (parents uneducated or kindergarten level, and temporary housing). One-third (32.5%) of students were exposed to SHS at home and most (66.5%) were exposed to SHS outside home in the past 7 days. Higher prevalence of medical consultation and hospitalisation was observed for students with exposure to SHS at home (P<0.001 for χ^2^ test) and outside home (P <0.001 for χ^2^ test) compared with students without respective exposures.

**Table 1:** Prevalence of medical consultation and hospitalisation by basic characteristics.

		Medical consultation in the past 14 days		Hospitalisation in the past 12 months	
		n = 5692, 15.9%		n = 1844, 5.2%	
	n (%)	%	χ^2^ P (value, df.)^a^	%	χ^2^ P (value, df.)^a^
Sex			<0.001		<0.001
Boys	16988 (47.5)	15.1	(16.6, 1)	5.9	(42.7, 1)
Girls	18795 (52.5)	16.6		4.4	
Age			0.22		0.03
≤15	16433 (45.9)	16.1	(1.3, 1)	5.4	(4.3, 1)
>15	19394 (54.1)	15.8		4.9	
Highest parental education			<0.001		<0.001
Unknown	5857 (17.8)	14.2	(50.3, 5)	4.5	(38.2, 5)
Uneducated or kindergarten	549 (1.7)	19.9		7.8	
Primary school	4181 (12.7)	15.3		4.8	
Form 1–3	7658 (23.3)	15.3		4.5	
Form 4–5	8274 (25.1)	15.8		5.3	
Form ≥6	6401 (19.4)	18.3		6.3	
Housing type			<0.001		<0.001
Public housing estate	14144 (43.2)	14.3	(88.8, 5)	4.4	(160.6, 5)
Private (subsidised)	3180 (9.7)	15.4		5.2	
Private (owner)	10461 (31.9)	17.2		5.0	
Private (tenant)	2765 (8.5)	16.5		6.0	
Temporary	340 (1.0)	28.8		18.8	
Others	1822 (5.6)	18.1		7.2	
SHS exposure at home			<0.001		<0.001
None	24180 (67.5)	15.2	(29.6, 2)	4.3	(104.5, 2)
1–4 days/week	5711 (15.9)	17.9		7.0	
5–7 days/week	5936 (16.6)	16.7		6.7	
SHS exposure outside home			<0.001		<0.001
None	11985 (33.5)	14.6	(53.2, 2)	4.4	(63.7, 2)
1–4 days/week	16732 (46.7)	15.7		4.9	
5–7 days/week	7110 (19.9)	23.2		7.0	

a χ^2^ value and degree of freedom.

### SHS exposure and medical services use

Among never-smokers, any SHS exposure at home was weakly associated with hospitalisation but not with medical consultation (Table 2). However, among current smokers, 1–4 and 5–7 days/week of exposure at home yielded AORs (95% CI) of 1.52 (0.97–2.38) and 2.05 (1.19–3.63) for medical consultation (P<0.01 for trend), and 3.23 (1.60–6.52) and 2.12 (0.88–5.00) for hospitalisation (P = 0.04 for trend), respectively, compared with 0 day of exposure. Any SHS exposure at home yielded AORs (95% CI) of 1.69 (1.14–2.51) for medical consultation and 2.85 (1.47–5.52) for hospitalisation in smokers. Robust corresponding AORs of 1.66 (1.11–2.48) and 2.78 (1.39–5.54) were observed after further adjusting for alcohol and drug use (not shown in tables). Medical consultation and hospitalisation were simultaneously reported by 2.3% of students, and such reporting was strongly associated with any SHS exposure at home among smokers (AOR 5.11, 95% CI: 2.10–12.47) (data not shown in tables). Moreover, increasing number of co-residing smokers was associated with AORs of 1.18 (1.00–1.41) for medical consultation and 1.33 (1.04–1.71) for hospitalisation among current smokers (data not shown in tables). The associations between the number of co-residing smokers and medical services use were significantly stronger in current smokers than never-smokers (P<0.01 for interaction). Non-significant findings were observed for SHS exposure outside home except for weak associations for medical consultation with AORs (95% CI) of 1.52 (1.00–2.29) for 5–7 days/week in current smokers and 1.19 (1.05–1.34) for 5–7 days/week and 1.08 (1.00–1.19) for any exposure outside home in never-smokers (P>0.05 for interaction).

**Table 2:** Adjusted odds ratios for SHS exposure and medical services use in current and never-smokers.

SHS exposure		Medical consultation in the past 14 days			Hospitalisation in the past 12 months		
day/week	n	%	Adjusted OR (95% CI)^a^	P for trend	%	Adjusted OR (95% CI)^a^	P for trend
At home^b^							
Current smokers							
None	344	18.3	1	<0.001	6.0	1	0.04
1–4	203	26.3	1.52 (0.97–2.38)		22.2	3.23 (1.60–6.52)	
5–7	101	29.6	2.05 (1.19–3.63)		14.3	2.12 (0.88–5.00)	
Any	304	27.4	1.69 (1.14–2.51)		19.6	2.85 (1.47–5.52)	
Never-smokers							
None	15113	14.0	1	0.84	3.7	1	0.08
1–4	2174	14.2	1.08 (0.94–1.24)		4.3	1.29 (1.00–1.66)	
5–7	1402	12.3	0.94 (0.78–1.13)		3.8	1.20 (0.89-1.61)	
Any	3576	13.4	1.03 (0.91–1.15)		4.1	1.25 (1.02–1.54)	
P for interaction^c^			<0.01			<0.01	
Outside home^d^							
Current smokers							
None	214	20.6	1	0.02	9.6	1	0.78
1–4	538	20.4	1.13 (0.74–1.72)		8.4	0.82 (0.39–1.72)	
5–7	578	24.4	1.52 (1.00–2.29)		10.3	1.00 (0.53–1.89)	
Any	1116	22.5	1.33 (0.89–1.96)		9.4	0.91 (0.48–1.73)	
Never-smokers							
None	9330	13.9	1	<0.01	3.9	1	0.29
1–4	10341	15.3	1.06 (0.97–1.17)		3.8	0.95 (0.83–1.08)	
5–7	2571	16.7	1.19 (1.05–1.34)		4.8	1.17 (0.95–1.45)	
Any	12912	15.5	1.08 (1.00–1.19)		4.0	0.99 (0.87–1.13)	
P for interaction^c^			0.24			0.81	

a Adjusting for sex, age, highest parental education, housing type, school clustering effects, mutually adjusted for SHS at home and outside home, and additionally adjusted for cigarette consumption per day and years of smoking in current smokers.

b Only students who had been exposed to SHS outside home ≤2 days/wk were included.

c P for interaction between any SHS exposure in current and never-smokers.

d Only students who had been exposed to SHS at home ≤2 days/wk were included.

## Discussion

Our study has provided the first evidence that SHS exposure at home was associated with medical services use in adolescent current smokers. The results were consistent with significant associations between the number of co-residing smoker and medical services use, and comparable to findings in many studies of never-smokers [2]–[4], [19], [20]. SHS exposure in smokers was associated with respiratory symptoms [6], [10], which were the main conditions requiring medical consultation among adolescents [21]. Our adolescent studies [22], [23] and other Western studies [24], [25] have also reported an association between SHS exposure and heavier smoking, which leads to illnesses. Our results showed that SHS exposure at home among adolescent smokers was far from trivial. Any SHS exposure at home was associated with excessive risks of 69% for medical consultation and 185% for hospitalisation among smokers. Health care providers should regularly assess SHS exposure among young patients, promote smokefree homes and help young patients quit smoking. Adolescent smokers, particularly those attending medical services, should be advised to be aware of the harm of SHS in addition to that from their own smoking. Parents should quit smoking or at least smoke outside to avoid exposing children to SHS at home. Public health interventions to promote smoking cessations and smokefree home in Hong Kong should be strengthened.

Compared with never-smokers, current smokers had greater odds of medical services use in relation to SHS exposure at home. Similarly, we have reported that the odds of respiratory symptoms due to SHS exposure at home was also greater in current smokers than never-smokers [10]. This might be due to the poorer health of smokers and more intensive SHS exposure. Contrary to the significant association between SHS exposure outside home and respiratory symptoms in adolescent smokers [10]. SHS exposure outside home was not significantly associated with medical services use. This might be due to the higher intensity of SHS exposure required to trigger medical services use among adolescents, especially smokers, who generally are reluctant to seek health even with apparent symptoms [26]. SHS exposure outside home was mainly from streets or restaurants [14], hence the intensity of exposure was probably lower than that at home. The time constraint in classrooms, typical of school-based surveys, did not allow us to collect detailed information about the causes of medical consultation and hospitalisation, and stronger associations are expected if analyses were restricted to smoking-related causes.

Like many Western cities, smoking has been banned in most public indoor places and workplaces in Hong Kong. The family home became the main place for smoking, and children were heavily exposed to SHS at home [14]. Children who smoke might be particularly at risk as parents would be less likely to avoid smoking in front of them than non-smoking children. Banning smoking in most indoor places inevitably clusters smokers in specific areas, rendering them a higher risk of SHS exposure. Smokers are probably more likely to smoke and be exposed to SHS at home. Further studies are warranted to investigate the effects of SHS exposure among smokers after the implementation of smokefree legislation in 2007.

This study is unique as SHS exposure is intense in this crowded city, which allowed testing for the under-studied effects of SHS exposure on smokers. However, our study has several limitations. All the data were self-reported and subjected to reporting bias. Smoking and SHS exposure were validated in another local youth smoking survey among 76 students and 66 never-smoking students. Satisfactory agreements of hair nicotine with self-reported smoking (83.5%) and SHS exposure (62.3%) were found. Candid reporting of smoking was encouraged using an anonymous questionnaire. Self-reported smoking and SHS exposure were also measures adopted by GYTS [16], [27]. Cotinine measures are more objective, but biomarkers may not distinguish passive smoking from active smoking or the place of exposure, which were the main factors in this study.

Although the use of medical services were self-reported, adolescents should have little difficulty in reporting such specific events especially hospitalisation. The validity was further supported by the observed significant associations of medical consultation and hospitalisation with health complaints and poor self-rated health (all P<0.05 for odd ratios). Smoking was significantly associated with higher odds of medical consultation (P<0.001) and hospitalisation (P<0.001). The association between SHS exposure and medical services use was not commonly perceived by students, and they were not aware that this was one of the objectives of the study. Any random misclassification of medical consultation and hospitalisation would have resulted in an underestimation of the effects. Reverse causality in that health services use prompted deliberate SHS exposure seems unlikely. If anything, it would lead to the avoidance of SHS and an underestimation of risk. Few smokers would be completely unexposed to SHS in a densely populated Hong Kong. Therefore, using 0 day/week of SHS exposure as the reference group might also have underestimated the risk. Lastly, although we have adjusted for many potential confounders (socio-demographic characteristics, smoking intensity and other risky behaviours) residual confounding cannot be excluded. We have no data on maternal smoking during pregnancy, which predicts smoking [28] and health services use [3] in children. However, given the low prevalence (3.6%) of smoking mother in China [29], the influence on the association, if any, should be small.

## Conclusions

SHS exposure at home was associated with health services use among adolescent current smokers. Adolescent smokers should be aware of the harm of SHS in addition to that from their own smoking. The evidence helps health care professionals to advise adolescent smokers to avoid SHS exposure and stop smoking.
